# Cystic fibrosis presentation in del. F508 and p. Tyr109Glyfs compound heterozygote *CFTR* state: a case report

**DOI:** 10.3325//cmj.2019.60.246

**Published:** 2019-06

**Authors:** Mirjana Turkalj, Vid Matišić, Arijana Šimić, Alen Juginović, Damir Erceg, Dorian Tješić Drinković, Wolfgang Höppner, Dragan Primorac

**Affiliations:** 1 Srebrnjak Children’s Hospital, Zagreb, Croatia; 2 School of Medicine, Josip Juraj Strossmayer University of Osijek, Osijek, Croatia; 3 Croatian Catholic University, Zagreb, Croatia; 4 University of Zagreb School of Medicine, Zagreb, Croatia; 5 School of Medicine, University of Split, Split, Croatia; 6 Faculty of Dental Medicine and Health, Josip Juraj Strossmayer University of Osijek, Osijek, Croatia; 7 Department of Pediatrics, University Hospital Center Zagreb, Zagreb, Croatia; 8 Bioglobe, Hamburg, Germany; 9 University of Hamburg, Hamburg, Germany; 10 Eberly College of Science, The Pennsylvania State University, University Park, PA, USA; 11 Henry C. Lee College of Criminal Justice and Forensic Sciences University of New Haven, New Haven, CT, USA; 12 St. Catherine Specialty Hospital, Zagreb/Zabok, Croatia; 13 Faculty of Medicine, University of Rijeka, Rijeka, Croatia

## Abstract

The diagnosis of cystic fibrosis (CF) is commonly confirmed by molecular genetics with the presence of specific mutations of *cystic fibrosis transmembrane conductance regulator* (*CFTR*) gene. We report a case of cystic fibrosis (CF) in a 15-year-old female patient who is a compound heterozygote for *CFTR* gene, with delta F508 and Tyr109Glyfs mutations detected. This is the first detailed description of such a case in the medical literature. The primary CF presentation occurred at the age of 9 in the form of gastrointestinal symptoms including greasy, bulky, and foul-smelling stool. The patient exhibited delayed growth, with her height and weight being below the 5th centile for age according to the World Health Organization growth curves. Pancreatic enzyme supplement treatment was started immediately, alongside high-fat and high-calorie diet, resulting in patient’s recovery and development. DNA analysis of *CFTR* gene demonstrated the presence of del. F508 mutation and a rare combining deletion and insertion mutation p. Tyr109Glyfs. The combination of the two mutations is very rare in CF patients and is therefore valuable to document this case in order to provide information on disease progression, therapy options, and outcomes. With standard treatment and early diagnosis, the patient is currently doing well and is not restricted by the disease in her daily and sports activities.

We report a case of a 15-year-old female patient with the clinical presentation of cystic fibrosis (CF) due to a rare compound heterozygote mutation. The combination of the two detected mutations and their impact on CF clinical presentation and progression is not described in the literature, with only 9 cases reported in the CFTR2 database (1).

## Case report

The patient’s first hospital admission was at the age of 9 because of poor growth, failure to gain weight even with increased food intake, and frequent stools (3-4 times a day). Her stools were periodically greasy, bulky, and strong-smelling. According to World Health Organization growth curves, the patient’s weight was in the 5th centile and height below the 5th centile for age. The patient’s birth history was unremarkable, with no early signs of CF. During the early infancy, the patient had recurrent upper respiratory tract infections, with constant cough and chronic nasal congestion treated with nebulized saline and occasionally oral antibiotics (when fever was present or expectorated mucus was purulent). Before her first visit, she had never had pneumonia and had not required hospital treatment. At the first visit, serum liver enzymes were elevated (aspartate aminotransferase 64 U/L, alanine aminotransferase 53 U/L, gamma-glutamyl transferase 35 U/L). Fat-soluble vitamins A, D, and E levels were decreased. Stool analysis detected fat droplets. Blood glucose levels were within the reference range. Blood coagulation parameters were just below the reference range. Inflammation parameters (eg, erythrocyte sedimentation rate, C-reactive protein, fibrinogen) were not increased. Abdominal ultrasound showed no abnormal findings. Ultrasound densitometry of both feet was normal. Pancreatic malabsorption was diagnosed by quantification of fecal elastase activity. The result was 15 µg/g, which was significantly low. The sweat test showed a high chloride level (104 mEq/L), which was confirmed when the test was repeated (107 mEq/L). Chest x-ray showed peribronchial thickening. Pulmonary function tests showed normal pulmonary function. Acid-base status showed slightly lowered arterial oxygen tension, with oxygen saturation of 96%-97%. Cystic fibrosis DNA testing was performed by Abbot CFv3 commercial laboratory test for 32 of the most common CFTR mutations, and concluded the patient was heterozygous for del. F508 mutation.

She was prescribed with pancreatic enzyme supplements (25 000 units/larger meal and 10 000 units/smaller meal) and fat-soluble vitamin supplements (vitamins A, D, E, K) to be taken daily together with a high-calorie, high-fat diet. With therapy, the stools normalized to 1-2 times a day and were only rarely greasy.

On one of her following visits, at the age of 12, she was coughing. Sputum analysis showed 25 polymorphonuclear cells with *Staphylococcus aureus* isolated. Oral azithromycin was prescribed for 5 days. Dornase alfa (human recombinant DNase) was also introduced. She was advised to do regular physical therapy at home. Control sputum analysis was done after therapy and it did not show any pathogens. Sputum samples for sputum microbiology were obtained on regular patients’ check-ups, and the analysis did not reveal pathogens.

She was monitored yearly with a modified 2-hour oral glucose tolerance test, with results within the reference range. Patient’s growth status improved, her weight was then in the 44th and her height in the 10th centile for age. She showed no signs of delayed puberty and had menarche at the age of 13, but her menstrual cycles were irregular due to malnutrition. Now at the age of 15, her weight is in the 56th and her height in the 19th centile for age.

**Figure 1:**
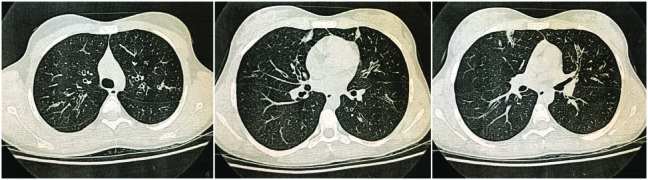
High-resolution computed tomography of the thorax showing ectatic bronchi. Thickened bronchial wall, with an impaction inside the lumen in both upper lung lobes, the middle lobe, and the lingula, is visible. Lymphadenopathy or pleural effusion is not visible.

Standard pulmonary function tests showed normal pulmonary function with forced expiratory volume 1 over 100% on multiple follow-ups, as well as forced vital capacity (FVC). Physical examination revealed minimal signs of nail clubbing. Chest CT showed ectatic bronchi, thickened bronchial wall, with an impaction inside the lumen in both upper lung lobes, the middle lobe, and the lingula (Figure 1).

On one of the recent check-ups, at age 15, she had symptoms of productive cough, especially in the morning. Physical examination revealed a few pulmonary base crackles. Chest x-ray showed acute bilateral hilar lymphadenopathy. Blood inflammation parameters were within the reference range. Bronchial aspirate analysis showed thick mucus with neutrophils, detritus, and bacteria: *Staphylococcus aureus*, *Pseudomonas aeruginosa*, and *Candida albicans*. Cardiac status was normal. She was prescribed oral ciprofloxacin and amoxicillin clavulanate. In post-therapy control sputum, *Pseudomonas aeruginosa* was isolated again. The patient is currently on aerosolized antibiotic therapy (tobramycin) at a 300 mg dose twice a day on alternate months for 6 months. She also uses saline nebulizer, positive pressure device to help mucociliary clearance, and high-frequency chest wall oscillation treatments.

She was examined by an ear, nose, and throat specialist. Fiberoptic endoscopy showed hypertrophic inferior nasal concha, bilateral middle nasal meatus polips, and a lot of serous discharge. Fluticasone propionate nasal drops and isotonized seawater spray were introduced in the therapy alongside dornase alfa. Nasal congestion symptoms have decreased significantly.

The patient has good school attendance records and is not restricted in her activities (dancing and tennis). Clinical CF expression was evident but we wanted to support the diagnosis by molecular genetics. Therefore, in May 2018 the patient's and parents’ blood was sent for additional genotyping of *CFTR* gene to BioGlobe, Hamburg, Germany (Table 1). The genotyping was performed by matrix-assisted laser desorption/ionization time-of-flight IPLEX technology and Sanger sequencing. The first mutation was del. F508 and the second was a combined deletion and insertion mutation. The three bases TAT were replaced by a G, resulting in a change from tyrosine in codon 109 to glycine and a subsequent stop codon due to frameshift. Parental genotypes showed that the father was a heterozygous carrier for the mutation delta F508, whereas the mother was a heterozygous carrier for the mutation c.325_327delTATinsG, TAT/G het p.(Tyr109Glyfs), confirming the diagnosis by molecular genetics.

**Table 1:** Gene analysis for 37 most common mutations of *CFTR* gene by matrix-assisted laser desorption/ionization time-of-flight IPLEX technology and Sanger sequencing.

	*CFTR* gene mutation	Comment
Father	del. F508	Heterozygous carrier of a CF*-causing mutation
Mother	c.325_327delTATinsG (p.Tyr109Glyfs)	Heterozygous carrier of a CF-causing mutation
Patient	• c.325_327delTATinsG (p.Tyr109Glyfs) • del. F508	Compound heterozygote for *CFTR* if the mutations are located on different parental chromosomes


*CF – cystic fibrosis.

## Discussion

The sequencing of patient’s and parents’ *CFTR* genotype confirmed the CF diagnosis, showing that our patient is a compound heterozygote with delta F508 and Tyr109Glyfs mutations detected. The two mutations acting in-trans result in the CF phenotype. Del. F508 is present in 70% of patients with CF (2), while p. Tyr109Glyfs is quite rare. It was first reported in a 3-year-old female patient in Slovenia with a severe form of the disease in 1993 and accounted for 1.1% of all the mutations found in 90 Hungarian CF patients in 2015 (3). Tyr109Glyfs mutation leads to a frameshift and terminates the translation at amino acid 112 of *CFTR* (4). The literature shows little data on these two mutations occurring together. CFTR2 database has records of only 9 patients with this gene combination (1). The average sweat chloride level in these 9 patients was 118 mEq/L, compared with 96 mEq/L in patients with two CF-causing variants. A total of 89% of patients with the rare mutations were suffering from pancreatic insufficiency, compared with 85% patients with two CF-causing variants. Furthermore, 44% of patients had *Pseudomonas* infection in comparison with 55% of patients with two CF-causing variants (1). There is insufficient lung function data for comparison. Although these results suggest that this rare mutation has poor clinical manifestations, our patient had a milder form of CF without severe lung infections and functions well in daily life, which is a novelty in itself and shows why early diagnosis and proper treatment is crucial when it comes to clinical outcomes. Compound heterozygosity involving the two mutations probably led to a somewhat less severe clinical manifestation of CF in our patient, which is rare when compared with other patients with these mutations. This confirms that phenotype-genotype determination of pathogenic mutations should be favored over genetic-phenotype determination of disease, especially because of scarcity of patients with this variant (1,5). We reinforce the recommendation for a wide mutation screening panel for patients with CF, considering the potential benefits of early recognition and treatment. Also, we strongly recommend to record useful patient data in databases like CFTR2 to ensure better treatment and diagnosis. Due to the paucity of literature information regarding patients with these two genetic variants, we believe that documenting and studying the case of this patient will provide information on therapy options and outcomes in future cases with the same genetic variant.
